# Transcatheter Aortic Valve Replacement in Young Low-Risk Patients With Severe Aortic Stenosis: A Review

**DOI:** 10.3389/fcvm.2020.608158

**Published:** 2020-12-14

**Authors:** Pier Paolo Bocchino, Filippo Angelini, Brunilda Alushi, Federico Conrotto, Giacomo Maria Cioffi, Gregorio Tersalvi, Gaetano Senatore, Giovanni Pedrazzini, Gaetano Maria De Ferrari, Luigi Biasco

**Affiliations:** ^1^Division of Cardiology, Department of Medical Sciences, University of Turin, “Città della Salute e della Scienza” Hospital, Turin, Italy; ^2^Department of General and Interventional Cardiology, Helios Klinikum Erfurt, Erfurt, Germany; ^3^Department of Cardiology, Campus Benjamin Franklin, Charite' Medical University Berlin, Berlin, Germany; ^4^Department of Cardiology, Kantonsspital Luzern, Lucerne, Switzerland; ^5^Division of Cardiology, Fondazione Cardiocentro Ticino, Lugano, Switzerland; ^6^Department of Internal Medicine, Hirslanden Klinik St. Anna, Lucerne, Switzerland; ^7^Azienda Sanitaria Locale Torino 4, Ospedale di Ciriè, Ciriè, Italy; ^8^Department of Biomedical Sciences, University of Italian Switzerland, Lugano, Switzerland

**Keywords:** transaortic valve replacement, TAVR, severe aortic stenosis, valvular heart disease, young patients, low risk

## Abstract

In the last decades, transcatheter aortic valve replacement (TAVR) revolutionized the treatment of symptomatic severe aortic stenosis. The efficacy and safety of TAVR were first proven in inoperable and high-risk patients. Then, subsequent randomized clinical trials showed non-inferiority of TAVR as compared to surgical aortic valve replacement also in intermediate- and low-risk populations. As TAVR was progressively studied and clinically used in lower-risk patients, issues were raised questioning its opportunity in a younger population with a longer life-expectancy. As long-term follow-up data mainly derive from old studies with early generation devices on high or intermediate surgical risk patients, results can hardly be extended to most of currently treated patients who often show a low surgical risk and are treated with newer generation prostheses. Thus, in this low-risk younger population, decision making is difficult due to the lack of supporting data. The aim of the present review is to revise current literature regarding TAVR in younger patients.

## Introduction

Aortic stenosis (AS) is the most common valvular heart disease in the adult population and its prevalence progressively increases according to population aging (1). Medical therapy yields poor outcomes in severe symptomatic AS treatment and surgical aortic valve replacement (SAVR) has long been considered the standard of care in suitable symptomatic patients with severe AS (2). The development of transcatheter aortic valve replacement (TAVR) provided a new strategy for their treatment that rapidly became a valid alternative approach in a progressively larger proportion of patients. From the interventional perspective, the procedure of TAVR has significantly evolved over the years. From the very first pioneering implant by Cribier, performed through a trans-venous, trans-septal, retrograde approach (3), nowadays procedural steps have been clearly defined. The percutaneous transfemoral approach performed under local anesthesia with conscious sedation is the most widely adopted in current practice, due to its easiness, low invasiveness and better short- and long-term outcomes (4, 5).

Aim of the present review is to revise the history of TAVR by focusing on its adoption in “young” patients and address current open issues regarding TAVR in this group.

## The Evolution of TAVR

### From Young to Old and Back to Young Patients

The first-in-man TAVR procedure was performed on a middle-aged patient. Back in 2002, Professor A. Cribier implanted a prototype balloon expandable valve on a 57-year-old man with severe calcific AS who was judged inoperable due to hemodynamic instability and significant associated comorbidities (3). The technical success of this procedure laid the foundation for subsequent studies investigating TAVR in inoperable and high-risk surgical candidates so as to offer a therapeutic option for such critical patients. When comparing the development of TAVR to the evolution of percutaneous coronary interventions it is clear that their development moved in opposite directions. While coronary interventions were first performed in extremely selected low-risk patients moving to higher-risk challenging cases (6), TAVR first focused on older, high/extreme surgical risk patients, and gradually approached younger and lower-risk subjects.

In this context, indication to TAVR was judged according to surgical risk scores such as the Society of Thoracic Surgeons (STS) score or the EuroScore (ES), both estimating the risk of 30-days mortality after open surgery.

According to the STS score, three categories of patients were identified, namely at high, intermediate or low risk (> 8%, 4–8%, or <4%, respectively), with only slight variations of the cut-off values among the studies or the less precise EuroScore II (7). Nevertheless, due to the limited numbers of criteria considered as variables in conventional surgical risk scores and their derivation from surgical series, the STS score and the EuroScore II have been proven to perform poorly in patients undergoing TAVR, thus highlighting the utmost role of appropriate clinical judgment in the choice between surgical vs. interventional approaches (8).

### The Evolution of TAVR: A Path Through Randomized Clinical Trials and Registries

The first randomized controlled trial on TAVR, the PARTNER 1B, was published in 2010 and compared TAVR with a balloon-expandable bovine pericardial Edwards SAPIEN Valve against medical therapy on extreme-risk patients (mean age 83.1 ± 8.6 years, mean STS score 11.2 ± 5.8). The trial reported a significantly lower rate of death at 1 year in the TAVR group compared to the medical treatment group, thus opening this therapeutic possibility (9). The evidence that a percutaneous treatment in non-surgical candidates was superior to standard medical therapy laid the ground for the subsequent PARTNER 1A trial, demonstrating the non-inferiority of TAVR vs. SAVR in high-risk patients (10). In parallel, the efficacy of the self-expandable Medtronic CoreValve bioprosthesis was demonstrated in non-operable as well as high-risk patients with the CoreValve Extreme risk study and the CoreValve High risk trial, respectively (11, 12).

In the following years, trials comparing, TAVR vs. SAVR on intermediate-risk patients demonstrated the non-inferiority of TAVR vs. SAVR with both the second generation balloon-expandable SAPIEN XT valve in the PARTNER 2A trial and the self-expandable CoreValve valve in the SURTAVI trial (13, 14). Recently, TAVR was evaluated in low-risk patients, with the PARTNER 3 trial showing the superiority of the balloon-expandable SAPIEN 3 valve compared to SAVR and the Evolut Low Risk Trial demonstrating non-inferiority against SAVR. (12, 15) Characteristics of the landmark TAVR trials are reported in Table 1.

**Table 1:** Characteristics of the landmark studies on transcatheter aortic valve replacement.

**Study**	**Year**	**Population**	**Arms**	**Age**	**Risk scores**	**Results**	**Complications**
**Extreme-risk**							
PARTNER B	2010	358 pts with severe AS, NYHA II-IV, STS > 50% or critical conditions	TAVR with Edwards SAPIEN valve vs. standard therapy (usually balloon aortic valvuloplasty)	83.1 ± 8.6	STS score 11.2% EuroScore 26.4%	TAVR significantly reduces the rate of all-cause death (HR 0.55), CV death (HR 0.39), death + rehospitalization (HR 0.46), symptoms and 6MWD at 1 yr and non-significantly increases major stroke rate at 30 days (5.0 vs. 1.1%, p 0.06) and 1 yr (7.8 vs. 3.9%, p 0.18).	Paravalvular aortic regurgitation 3–4+ in TAVR: 11% at 30 days and 1 yr
CoreValve Extreme Risk	2014	489 pts with severe AS, NYHA II-IV, STS > 50% or irreversible 30-days SAVR morbidity	TAVR with CoreValve vs. OPG (43% for all-cause death or stroke at 1 yr)	83.2 ± 8.7	STS score 10.3% EuroScore 22.6%	All-cause death or stroke at 1 yr 26.0% (upper 95%CI 29.9% < OPG 43%). Significant improvement in NYHA class	PM implantation: 21.6% at 30 days, 26.2% at 1 yr. Paravalvular aortic regurgitation 3–4+: 4% at 1 yr
**High-risk**							
PARTNER A	2011	699 pts with severe AS, NYHA II-IV, STS > 10%	TAVR with Edwards SAPIEN valve vs. SAVR	83.6 ± 6.8	STS score 11.8% EuroScore 29.3%	No difference in all-cause death at 1 yr (24.2% TAVR vs. 26.8% SAVR). More neurological events (strokes + TIA) in TAVR pts at 30 days and 1 yr (p 0.04). Shorter hospital stay and shorter ICU stay in TAVR patients	Higher rate of vascular complications at 30 days in TAVR. No difference in new PM implantation (4%). More major bleeding events and new-onset AF in SAVR
CoreValve High Risk	2014	747 pts with severe AS, NYHA II-IV, STS 15–50%	TAVR with CoreValve vs. SAVR	83.2 ± 7.1	STS score 7.3% EuroScore 17.6%	TAVR reduces 1 yr all-cause death (14 vs. 19%, p 0.04) and neurological events (20 vs. 27%)	More PM implantations and major vascular complications in TAVR. More bleeding events, AKI and new or worsening AF in SAVR
**Intermediate-risk**							
PARTNER 2A	2016	2,032 pts with severe AS, NYHA II-IV, STS 4.0–8.0%	TAVR with SAPIEN XT vs. SAVR	81.5 ± 6.7	STS score 5.8% EuroScore *N/A*	No difference in primary composite endpoint (all-cause death + disabling stroke) at 2 yrs; similar single components of the primary endpoint. TF TAVR reduces the primary endpoint (HR 0.78, p 0.05)	More major vascular complications and paravalvular aortic regurgitation but less life-threatening bleeding, AKI, and new-onset AF in TAVR. Similar new PM implantation rates (8.5 vs. 6.9%, p 0.17)
SURTAVI	2017	1,160 pts with severe AS, NYHA II-IV, STS 3–15% and non-traditional risk factors	TAVR with CoreValve *or* Evolut R vs. SAVR	79.8 ± 6.2	STS score 4.4% EuroScore 11.9%	TAVR non-inferior for composite primary endpoint (all-cause death + disabling stroke) at 2 yrs. No structural valve deterioration at 2 yrs in both groups	Higher aortic regurgitation (5%) and new PM implantation (26 vs. 7%) in TAVR. Higher AKI (4%), AF (43%) and blood transfusions in SAVR
**Low-risk**							
NOTION	2015	280 pts ≥ 70 years-old with severe AS	TAVR with CoreValve vs. SAVR	79.2 ± 4.9	STS score 2.9% EuroScore 8.4%	TAVR non-inferior for composite primary endpoint (all-cause death + stroke + MI) at 1 yr	TAVR showed fewer post-operative major bleedings, cardiogenic shock, AKI and new AF, and shorter hospital stay. At 1 yr, TAVR showed more PM implantations, total aortic regurgitation, and higher NYHA class
Evolut Low Risk	2019	1,403 pts with severe AS, NYHA II-IV, STS < 3%	CoreValve *or* Evolut R *or* Evolut PRO TAVR vs. SAVR	74.0 ± 5.9	STS score 1.9% EuroScore *N/A*	TAVR non-inferior for composite primary endpoint (all-cause death + disabling stroke) at 2 yrs	TAVR shows fewer 30-days disabling strokes, bleeding complications, AKI, and AF but higher 3–4+ aortic regurgitation (3.5%) and new PM implantation rates (17%)
PARTNER 3	2019	1,000 pts with severe AS, NYHA II-IV, or asymptomatic pts with ejection fraction < 50%, STS < 4%	TF TAVR with SAPIEN 3 vs. SAVR.	73.3 ± 5.8	STS score 1.9% EuroScore II 1.5%	TAVR non-inferior for composite of all-cause death, stroke, and rehospitalizations at 2 yrs (HR 0.54, p 0.001). TAVI shows lower 30-days stroke (HR 0.25), death or stroke (HR 0.30) and new AF (HR 0.10), shorter index hospitalization, fewer major bleeding events (HR 0.12) but more new left bundle branch block (HR 3.43)	No difference in major vascular complications and new PM implantation rates at 30 days, nor in 3–4+ aortic regurgitation at 30 days or 1 yr
UK TAVI	2020	913 pts with severe AS, aged ≥ 80 yrs or ≥ 70 yrs with intermediate/high surgical risk	TAVR vs. SAVR	81.1 ± 4.4	STS score 2.6% EuroScore *N/A*	TAVR non-inferior for all-cause death (HR 1.91, p 0.33), CV death (HR 2.22, p 0.27), stroke (HR 0.95, p 0.93), or the composite of all-cause death + stroke (HR 0.95, p 0.88) at 1 yr compared to SAVR	TAVR is associated with less major bleeding, shorter hospital stay, and more rapid improvement in NYHA class and QoL as well as more vascular complications, PM implantations, and mild or moderate aortic regurgitation than SAVR.
**Direct comparison**							
CHOICE	2014	241 pts with severe AS, NYHA II-IV, high surgical risk (≥ 75 yo or STS ≥ 10% or EuroScore ≥ 20%), suitable for TF access and contraindication to SAVR	TF TAVR with a first-generation balloon-expandable valve vs. first-generation self-expanding valve	81.9 ± 6.7	STS score 5.6% EuroScore 21.5%	Device success was more frequent in the balloon-expandable group compared to the self-expanding group (RR 1.24, *p* < 0.001).	Significantly lower moderate to severe aortic regurgitation, less frequent need for implanting more than 1 valve, and fewer PM implantations in the balloon-expandable valve group. Cardiovascular mortality and bleeding and vascular complications were not significantly different
SCOPE	2019	739 pts ≥ 75 yo with symptomatic severe AS at increased surgical risk	ACURATE neo *vs* SAPIEN 3 TAVR systems	82.8 ± 4.1	STS score 3.5% EuroScore *N/A*	Non-inferiority of the ACURATE neo was not met as for the primary composite efficacy and safety endpoint (absolute risk difference 7.1%, p 0.42) at 30 days compared to SAPIEN 3.	More frequent AKI and moderate or severe aortic regurgitation with ACURATE neo. No difference in all-cause death and stroke between the study groups.
SCOPE II	2020	796 pts ≥ 75 yo with indication to transfemoral TAVR	ACURATE neo *vs* Evolut R TAVR systems	83.2 ± 4.3	4.6 ± 2.9%	Non-inferiority of the ACURATE neo was not met as for the primary composite endpoint (absolute risk difference 1.8%; p=0.0549) at 1 year compared to Evolut R	More frequent cardiac deaths, moderate or severe aortic regurgitation at 30 days while lower PM implant rate were observed in the ACURATE neo group
SOLVE-TAVI	2020	438 pts ≥ 75 yo, severe AS, NYHA II-IV, STS>10%, or Euro Score>20%	TF TAVR with SAPIEN 3 vs. Evolut R	81.7 ± 5.3	STS score 4.7% EuroScore 15%	No difference in composite of all-cause death, stroke, 3–4+ paravalvular leak and permanent PM implantation at 30 days (28.4 vs. 26.1%, p 0.04 for equivalence). No difference in individual components of the primary endpoint	PM implantation rates SAPIEN 3 19% vs. Evolut R 23% (p 0.06 for equivalence). Numerically higher strokes with SAPIEN 3 (4.7 vs. 0.5%, p 0.003 for equivalence)

*6MWD, 6-min walking distance; AF, atrial fibrillation; AKI, acute kidney injury; AS, aortic stenosis; CI, confidence interval; CV, cardiovascular; HR, hazard ratio; ICU, intensive care unit; MI, myocardial infarction; N/A, not available; NYHA, New York Heart Association; pts, patients; OPG, objective performance goal; PM, pace maker; QoL, quality of life; SAVR, surgical aortic valve replacement; STS, Society of Thoracic Surgeons; TF, transfemoral; TIA, transient ischemic attack; yo, years-old; yr, year*.

The consistent reduction of the estimated surgical risk throughout the years in trials' populations paralleled the reduction in the mean age (with an inverse increase in expected survival) of patients enrolled in the different trials (from mean age 83.1 ± 8.6 years in the PARTNER IB to mean age 74.0 ± 5.9 years in the Evolut Low Risk Trial), as age represents one of the main determinants of surgical risk. This trend clearly urges to consider several aspects of TAVR, such as procedural safety, long term efficacy, freedom from reinterventions, and patient's expectations, all of which have tremendous impact when evaluating different therapeutic options in young patients with severe AS.

The outspread performance of TAVR procedures boosted by the promising trials' results fueled the birth of national registries, providing encouraging TAVR data on wider populations in real-life scenarios (16–21). The progressive trend in lowering of the surgical risk of the TAVR patients included in randomized trials was also evident in real life all-comers registries (18, 20). When only low-risk patients were considered, the mean logistic EuroScore decreased down to 10.8% (21). Overall, the mean age of patients enrolled in TAVR registries remained stable at about 80–82 years across all reports, but more attention has been recently given to younger patients, with latest data focusing specifically on this peculiar population (22). Basic features of TAVR registries are reported in Table 2.

**Table 2:** Characteristics of the published registries on transcatheter aortic valve replacement.

**Registry**	**Year**	**Country**	**Population**	**Age**	**Risk scores**	**Results**	**Complications**
FRANCE 2	2012	France	4,165 pts undergoing TAVR between 2010 and 2012	82.8 ± 7.1	EuroScore 21.7%	In-hospital mortality rate 8.1% and 30-days mortality rate 10.1%. Discharge by day 5 post-TAVR 11.9%	Stroke rate 2.0%. Permanent PM implantation 12.6%. Paravalvular aortic regurgitation 3–4+ 15.7%
FRANCE TAVI	2017	France	12,804 pts who underwent TAVR for severe AS between 2013 and 2015	83.4 ± 7.2	EuroScore 17.9% (*p* < 0.001)^*^	In-hospital mortality rate 4.9 % (*p* < 0.001)^*^ and 30-days mortality rate 5.4% (*p* < 0.001)^*^. Discharge by day 5 post-TAVR 24.7% (*p* < 0.001)^*^	Stroke rate 2.0% (p 0.82)^*^. Permanent PM implantation 17.5% (*p* < 0.001)^*^. Paravalvular aortic regurgitation 3–4+ 10.2% (*p* < 0.001)^*^
GARY - intermediate risk	2018	Germany	7,613 pts undergoing isolated TAVR or SAVR for severe AS between 2012 and 2014, STS 4.0–8.0%	82.5 ± 5.0	STS score 5.6% EuroScore 21.2%	No difference in in-hospital mortality (3.6 vs. 3.6%, p 0.976) and propensity-matched 1-year mortality (17.1 vs. 15.7%, p 0.59) between TAVR and SAVR	No difference in in-hospital major stroke (1.4 vs. 1.0%, p 0.201) between TAVR and SAVR. More new PM (18.1 vs. 4.0%, *p* < 0.001), vascular complications (7.8 vs. 0.9%, *p* < 0.001) and aortic regurgitation ≥2+ (4.3 vs. 0.5%, *p* < 0.001) in TAVR. Higher rate of postprocedural bleeding requiring transfusion (22.1 vs. 59.6%, *p* < 0.001) or reintervention (1.0 vs. 4.7%, *p* < 0.001) and temporary dialysis (1.8 vs. 6.5%, *p* < 0.001) in SAVR.
SWISS TAVI	2018	Switzerland	4,599 pts undergoing TAVR between 2011 and 2016	82.2 ± 6.3	STS score 5.5% EuroScore 18.2%	30-days mortality 3.8% and 1-year all-cause mortality 13% and cardiovascular mortality 9.0%	30-days new PM 18.5%, AKI 4.8%, disabling stroke 1.9%, major bleeding 7.3%. 1-year new PM 20.2%, MI 0.9%, disabling stroke 2.6%, major bleeding 8.3%
UK TAVI	2018	United Kingdom	13,198 pts undergoing TAVR between 2007 and 2016	82	EuroScore 18.5%	Decline in in-hospital mortality from 4.7% in 2013 down to 1.8% in 2016	Stroke rate 2%, dyalisis requirement <1% in 2016
AQUA	2018	Germany	6,974 pts aged 65–74 yrs undergoing TF-TAVR or isolated SAVR in 2013 and 2014	71.6 ± 2.5	EuroScore 12.2%	After propensity-matching, no difference in in-hospital mortality (1.3 vs. 1.9%, p 0.39), stroke/TIA (1.0 vs. 2.1%, p 0.09) and MI (0 vs. 0.3%, p 0.16) between TF-TAVR and SAVR. Shorter postoperative hospital stay after TF-TAVR (9.5 vs. 12.5 days after SAVR, *p* < 0.001)	More frequent PM implantation after TF-TAVR (13.3 vs. 3.5%, *p* < 0.001). More frequent postoperative delirium after SAVR (8.9 vs. 2.4%, *p* < 0.001).
GARY –low risk	2019	Germany	20,549 pts undergoing isolated TAVR or SAVR for severe AS in 2014 and 2015, STS < 4.0%	78.9 ± 5.6	STS score 2.9% EuroScore 12.9%	Higher in-hospital and 30-days survival in TAVR patients than SAVR (98.5 vs. 97.3%, *p* = 0.003; 98.1 vs. 97.1%, *p* = 0.014). No difference in 1-year survival (90.0 vs. 91.2%, *p* = 0.158)	TAVR associated with more frequent PM implantation (15.1 vs. 4.4%, *p* < 0.0001), vascular complications (2.2 vs. 0.5%, *p* < 0.0001) and aortic valve regurgitation ≥2+ (3.0 vs. 0.5%, *p* < 0.0001)
OBSERVANT	2019	Italy	4,801 pts aged < 80 yrs undergoing TAVR or SAVR for severe AS between 2010 and 2012	75.0 ± 2.1	EuroScore 10.8%	5-years TAVR mortality 65.2% in pts < 65 yo, 48.5% in pts 65–74 yo, 55.2% in pts 75–79 yo. 5-years SAVR mortality 9.3% in pts < 65 yo, 15.9% in pts 65–74 yo, 23.7% in pts 75–79 yo	After TAVR: stroke 0.0% in pts < 65 yo, 1.3% in pts 65–74 yo, 2.0% in pts 75–79 yo; new PM 13.0% in pts < 65 yo, 13.9% in pts 65–74 yo, 16.7% in pts 75–79 yo; AKI 4.4% in pts < 65 yo, 4.1% in pts 65–74 yo, 7.2% in pts 75–79 yo; need for blood transfusions 34.8% in pts < 65 yo, 33.3% in pts 65–74 yo, 31.1% in pts 75–79 yo
YOUNG TAVR	2019	Germany, Italy, Poland	1,002 pts undergoing TF TAVR between 2013 and 2016	*N/A* overall	STS score: - 4.1% (<75 yo)−5.7% (76–86 yo)−8.7% (> 86 yo)	No difference in all-cause mortality between intermediate-age and older vs. younger groups at 30 days (HR 0.76, p 0.37 and HR 1.27, p 0.51, respectively) and 1 yr (HR 0.72, p 0.12 and HR 1.11, p 0.34, resepectively). Lower 2 yrs all-cause mortality in intermediate group compared to younger group (HR 0.62, p 0.01), but no difference between older and younger groups (HR 1.06, p 0.79)	Significantly lower risk of MACE in the intermediate-age group compared to the younger group at 2 yrs.

* *p-values are reported in comparison to the results of the FRANCE 2 registry. Abbreviations as in Table 1*.

While only few studies specifically focused on younger patients, current randomized trials enrolled patients according their high or intermediate surgical risk, thus intrinsically selecting elderly patients. This is evident when considering that mean age only decreased from 83 years in the PARTNER 1B trial to 73 years in the PARTNER 3 trial despite a concomitant drop in STS score from 11.2% down to 1.9% (9, 15).

Beside age and surgical risk, several clinical parameters, such as myocardial fibrosis, renal dysfunction, diabetes mellitus and pulmonary hypertension, have been identified as predictors short term all-cause mortality (23–25).

While only, preliminary evidence is currently available supporting the treatment of younger patients, patients requests for a percutaneous treatments in particular in younger subjects are becoming a matter of daily negotiation. The initial data deriving from the AQUA registry, addressing symptomatic patients younger than 75 years-old with an average intermediate surgical risk (EuroScore 12.2%), reported similar in-hospital outcomes for TAVR and SAVR with the exception of a more frequent need for new pacemaker implantation and a less frequent incidence of postoperative dialysis and delirium in TAVR (22).

Beside the progressive extension of TAVR to lower risk, younger populations, current research is focused on head to head comparisons between different transcatheter devices in order to ascertain the best fit between patient and current commercially available prostheses. Table 1 reports details on currently available direct comparisons between different devices (26–29). The results of recent trials support the safe implantation of newer generation valves, which have the advantage of better adaptation to the individual valve anatomy (26).

## It Is Never too Late to Be Young: TAVR in “Young” Patients

**Figure 1:**
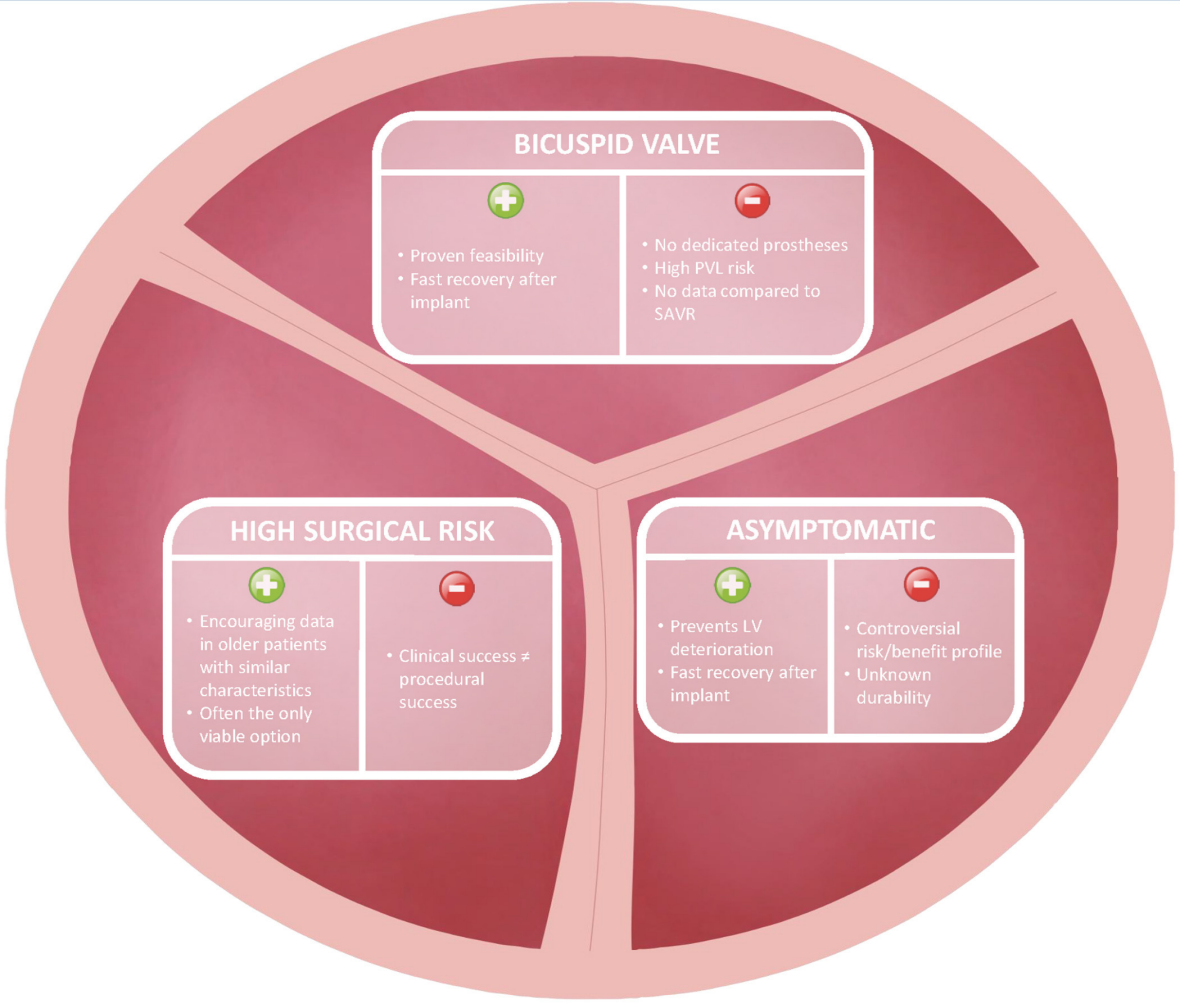
Pros and cons of transcatheter aortic valve replacement in young patients.

Apart from rare congenital cardiovascular diseases, severe AS is commonly diagnosed in middle-aged and elderly patients. By “young” AS patients we define individuals between 60 and 75 years of age, who have been only marginally included in current TAVR trials but might personally prefer or deserve percutaneous approach according to peculiar clinical characteristics. In our practice, we encountered three different groups of young patients in whom TAVR might be considered as an alternative to surgery, namely asymptomatic active patients with degenerative AS on a tricuspid valve, severe AS patients on a bicuspid aortic valve and severely comorbid “young” patients with severe AS unsuitable for the traditional surgical approach (i.e., the “Cribier's patient”) (Figure 1).

### Asymptomatic Patients

As new severe AS therapy opportunities were put forward and guidelines indications were updated, doubts and controversies were raised regarding the treatment of asymptomatic patients (1). While no data are yet available to support the treatment of asymptomatic AS patients with TAVR, some evidence is becoming available for SAVR. The recent RECOVERY trial compared early SAVR vs. conservative management in asymptomatic patients with severe AS (mean age 63.4 ± 10.7 years) and highlighted a significantly lower incidence of operative mortality or cardiovascular death in the early intervention group (30). The mean age of patients included in this trial clearly highlights that severe AS is not unique to elderly patients. Likewise, propensity-matched data from CURRENT AS registry stress that early aortic valve replacement (98% SAVR, mean age 71.6 ± 8.7 years) provides better 5-years survival and fewer heart failure hospitalizations compared to conservative strategies (31). Following the RECOVERY trial and the CURRENT AS registry, a meta-analysis encompassing a total of 29 observational studies with 4,075 patients showed high rates of all-cause death, cardiac death and progression to aortic valve intervention in untreated patients and demonstrated that early AS intervention was associated with a significant reduction in long-term mortality (HR 0.38; 95%CI 0.25–0.58) compared to a conservative strategy (32). These studies mostly comprised SAVR-treated patients in the intervention group. If similar results were confirmed with a percutaneous approach, it would pave the road to a paradigm shift from a symptomatic approach to “prophylactic” treatment of severe AS in the absence of symptoms.

It is common knowledge that a certain number of asymptomatic severe AS patients are currently treated with TAVR despite the lack of strong supporting evidence derived from dedicated studies. The paucity of limiting symptoms and their relative younger age usually allow these patients to be still professionally or physically active. In addition, asymptomatic subjects might have reduced perception of the valvular disease, thus preferring or accepting only a percutaneous solution able to shorten their absence from professional and personal duties as compared to conventional surgery. Clearly, when referring asymptomatic patients to TAVR procedural safety and long-term efficacy must be warranted in order not to dissipate its clinical benefit.

Research is moving faster to address unresolved issues regarding young AS patients. From a physiological and structural point of view, myocardial fibrosis represents a key progressive phase in left ventricular hypertrophic response to severe AS and its detection at late gadolinium enhancement cardiac magnetic resonance was proved to be an independent predictor of all-cause mortality in patients with AS (25). The EVoLVeD trial (NCT03094143) is currently enrolling asymptomatic severe AS patients older than 18 years old, with mid-wall late gadolinium enhancement as an early marker of left ventricle decompensation, to assess if early SAVR would improve the composite outcome of all-cause mortality and AS-related hospitalizations (33). Other interesting insights are expected from the EARLY-TAVR trial (NCT03042104), which started enrollment in 2017 and is randomizing asymptomatic patients aged 65 years or older to undergo either TAVR with the SAPIEN 3 bioprosthesis or clinical surveillance. Asymptomatic patients are likely bound to represent the next frontier for TAVR in the upcoming years.

### Bicuspid Aortic Valve

Severe AS on a bicuspid aortic valve (BAV) shows unique features such as presentation at an earlier age and technical issues related to peculiar anatomical characteristics. The first landmark trial on TAVR addressing BAV patients was published by Mylotte in 2014 (mean age 78.0 ± 8.9 years), demonstrating the feasibility of a percutaneous approach with promising outcomes on the short- and intermediate-term, albeit with a higher incidence of post-implantation aortic regurgitation mainly due to a more complex anatomy compared to tricuspid valves (34). Notably, although no differences between patients treated with TAVR with either a bicuspid or tricuspid anatomy were evident at 30 day (35), higher bailout TAVR-in-TAVR and lower device success rates were reported in BAV patients; indeed, careful patient selection, and anatomical assessment are paramount to warrant procedural safety and long-term efficacy (36).

Based on the STS/American College of Cardiology TVT Registry, including 81,822 consecutive patients with AS undergoing TAVR, Makkar et al. compared 2,691 BAV patients to a matched cohort of tricuspid aortic valve patients (median age 74 years, mean STS score 5%) (37). Similar 30-day (2.6 vs. 2.5%) and 1-year mortality (10.5 vs. 12.0%) rates and incidence of moderate/severe paravalvular leak at 30 days and 1 year were reported, alongside an increased 30-day risk for stroke in patients with BAV (2.5 vs. 1.6%) (37).

The recently published BEAT registry compared 242 BAV patients treated with Sapien 3 vs. 111 patients treated with Evolut R/PRO (mean age 77.8 years, mean STS score 4.4%) and confirmed the good procedural results with both platforms but with a higher rate of moderate-sever paravalvular aortic regurgitation at 1 year for the Evolut R/PRO group and a more frequent occurrence of annular rupture with balloon-expandable valves (38). As a result, the presence of a BAV, mainly due to the associated ellipticity of the aortic annulus, has a definite impact of the prosthesis' choice, as showed by a recent survey, with current evidences supporting the adoption of a self-expandable device (39).

### Young Patients at High Surgical Risk

TAVR use is increasing rapidly among young adult patients in recent years (40). Although TAVR may be an appealing option for young adults with severe AS in general due to faster recovery, avoidance of a chest scar, no need for general anesthesia, in certain subgroups of young but frail patients TAVR might *de facto* be the only feasible option due to their high surgical risk (38). To investigate the risk profile and baseline clinical characteristics of young patients undergoing percutaneous or surgical treatments for severe symptomatic AS, the OBSERVANT study analyzed data on 4,801 patients younger than 80 years undergoing isolated TAVR or SAVR (21). The study reported a remarkable difference in the clinical characteristics of patients undergoing TAVR compared to those undergoing SAVR, with the logistic EuroScore being significantly higher in TAVR patients as compared to SAVR among all age subgroups (7.90 vs. 2.40% in patients < 65 years, 10.57 vs. 4.91% in those aged 65–74 years and 11.19 vs. 7.52% in patients aged 75–79 years, respectively). Moreover, TAVR patients younger than 65 years showed the highest short and long term mortality as compared to older patients, mainly due to higher baseline surgical risk and frailty rather than procedural complications (21).

The YOUNG TAVR multicenter registry described characteristics and mortality after TAVR in different age groups (41). Patients aged 75 years or less had significantly higher rates of chronic obstructive pulmonary disease, diabetes and coronary artery disease, lower ejection fraction. and slightly higher left ventricular end-diastolic diameter compared to intermediate (76–86 years) and older (more than 86 years) age groups, despite having a significantly lower estimated STS 30-days mortality score (4.11 vs. 5.65 vs. 8.65% in younger, intermediate and older age groups, respectively; *p* < 0.001). Compared with younger patients, intermediate-age and older patients showed no difference in 30-day and 1-year all-cause mortality, but the intermediate age group showed lower all-cause mortality at 2 years (HR, 0.62; *p* = 0.01). Thus, according to clinical experience and YOUNG TAVR registry findings, “young” patients currently treated with TAVR are a unique subgroup with peculiar comorbidities impacting on mid-term outcomes. Their surgical risk, however, remains only partially captured by surgical risk scores, as age still plays a pivotal role in the STS risk assessment (41).

These data outline that not all young patients carry a low surgical risk but this group might hide patients that are not suitable for any other option, as in the case of the first patients treated by Professor Cribier. Moreover, the mortality risk assessment by means of the STS tool might be inadequate in this young patients' cohort. As a result, careful patient selection and clinical assessment for TAVR treatment must be warranted in this population to identify higher-risk individuals and guarantee a patient-tailored treatment.

### Unanswered Questions: Valve Durability, Conduction Disturbances, and Coronary Access

The overall encouraging and reassuring results of TAVR trials in lower risk, younger patients must be faced against the risk of procedural complications and unanswered questions coming with the TAVR procedures.

TAVR durability and efficacy in younger patients with fewer comorbidities and longer life-expectancy have been questioned (42). TAVR long-term follow-up data mainly derive from studies performing TAVR with early generation devices on high or intermediate surgical risk patients and results are hardly extendable to current low-risk patients with newer valve prostheses. For instance, the NOTION trial randomized low surgical risk patients (mean age 79.4 years) with severe AS to TAVR or SAVR in a 1:1 fashion and described similar rates of all-cause mortality at 6 years (42.5 vs. 37.7%, respectively, *P* = 0.58) with higher rates of structural valve deterioration for SAVR than TAVR (24.0 vs. 4.8%, respectively; *P* < 0.001) (43). Nevertheless, all TAVR patients received a CoreValve bioprosthesis and results may hardly be generalizable to balloon-expandable or newer-generation self-expanding valves. Long-term outcomes of the PARTNER 1 trial demonstrated similar all-cause mortality at 5-years follow-up for TAVR and SAVR (67.8 vs. 62.4%; *P* = 0.76) with higher rates of moderate or severe aortic regurgitation in the TAVR group compared to SAVR (14 vs. 1%; *P* < 0.0001), which was associated with increased 5-years risk of all-cause death in the TAVR group (44). A recent meta-analysis on structural durability of TAVR with both balloon-expandable and self-expanding valves vs. SAVR showed high rates of paravalvular regurgitation, moderate or severe aortic regurgitation, and reintervention at 5-years follow-up in the TAVR group compared to SAVR, thus highlighting that valve deterioration is still one of the open issues of percutaneous devices (45). Again, data were derived from trials using earlier generation TAVR valves in higher surgical risk (46, 47). Bioprosthetic valve failure and severe structural valve deterioration rates have been estimated to be around 4.6 and 1.3% at long-term follow-up, respectively (48).

The clinical impact of conduction disturbances after TAVR has been largely debated as well. High grade atrio-ventricular block and new-onset left bundle branch block are the most frequent complications following TAVR (49). Apart from the mechanical interaction between the prosthesis and the conduction system, there is evidence supporting the association between severe aortic stenosis itself, causing calcium deposition on the conduction system and the development of left ventricular dysfunction, and conduction disturbances.

The 2020 SOLVE-TAVI trial comparing newer generation self-expanding vs. balloon-expandable valves in TAVR procedures showed a near-significant trend toward higher permanent pacemaker implantation rate at 30 days in the self-expanding group and compared to the balloon-expanding group (23.0 vs. 19.2%, *P* = 0.06) (26). A meta-analysis of 30 studies reported that new-onset persistent left bundle branch block and permanent pacemaker implantation after TAVR are associated with an increased risk of death and heart failure hospitalization at 1 year, while periprocedural new-onset left bundle branch block was also associated with higher cardiac mortality and pacemaker implantation rates within the year following the procedure (50). As morbidity and mortality associated with these conditions are not trivial, careful patient evaluation and selection for TAVR procedures, in particular in the younger patients subgroups, are paramount (51).

TAVR assessment in younger patients must also take into account that their longer life expectancy might require future percutaneous procedures, either coronary or valvular or both. This undoubtedly impacts on valve selection, as prostheses allowing rapid and effective access to the coronary arteries or eventual valve-in-valve TAVR shall be preferred (39). Due to the peculiar design of most prostheses, current self-expandable devices might hamper the access to coronary ostia and thus limit the possibility of future percutaneous coronary interventions, which comes as a relevant prognostic drawback in the setting of acute coronary syndromes.

## Conclusions

Adoption of a percutaneous approach for the treatment of severe AS in progressively younger patients is one of the latest trends in contemporary interventional cardiology. As detailed in our review, different subgroups of patients with their peculiar clinical characteristics are currently encountered in clinical practice. In particular, the paradigm shift from a symptomatic to a “prophylactic” treatment of severe AS represents one of the major challenges of modern cardiology, radically changing the decision-making process in the treatment of valvular heart diseases. While opening newer therapeutic possibilities and potentially amplifying the prognostic approach of surgical and percutaneous treatments, this paradigm shift will challenge operators to achieve absolute procedural safety and ascertained long-term efficacy.

As severe AS is a complex pathology requiring comprehensive clinical and instrumental multidisciplinary evaluation and to date, no single algorithm can provide a patient-tailored approach, the Heart Team plays an essential role in deciding the optimal treatment strategy, especially when treating young patients with scarce data guiding the decision-making process.

## Author Contributions

PPB, FA, BA, FC, and LB conceived the study and carried out the first draft of the manuscript and its major revisions. GC, GT, GS, GP, and GMDF revised and edited the manuscript. All authors contributed to the article and approved the submitted version.

## Conflict of Interest

The authors declare that the research was conducted in the absence of any commercial or financial relationships that could be construed as a potential conflict of interest.
